# A space-time analysis of recurrent malnutrition-related hospitalisations in Kilifi, Kenya for children under-5 years

**DOI:** 10.1186/s40795-019-0296-5

**Published:** 2019-06-04

**Authors:** Kennedy Mwai Wambui, Eustasius Musenge

**Affiliations:** Division of Epidemiology and Biostatistics, School of Public Health, Faculty of Health Sciences, University of the Witwatersrand, Johannesburg, South Africa

**Keywords:** Spatiotemporal analysis, Child malnutrition, Bayesian, Child mortality

## Abstract

**Background:**

Globally, malnutrition underlies 45% of under-5 s mortality, mainly from potentiating common infections such as diarrhoea and pneumonia. Malnutrition as a public health problem is not evenly disbursed because of disparities in food insecurity and health, and children commonly suffer recurrent episodes of opportunistic infections. We aimed to understand better the spatial and temporal structure of multiple paediatric hospital admissions associated with malnutrition-related illnesses. This paper aimed to investigate the spatial-temporal variations in malnutrition-related recurrent morbidity of children under-5 years from the Kilifi County in Kenya between 2002 and 2015.

**Methods:**

The study included data from children under-5 years old who had more than one admission to a rural district hospital in Kenya within the Kilifi Health and Demographic Surveillance System (KHDSS). The primary outcome was a malnutrition-related admission, based on wasting (WHZ < -2) or nutritional oedema. Individual, household and environmental level covariates were examined as exposures. We first fitted a SARIMA model for the temporality, and the Moran’s Index affirmed spatial clustering in malnutrition admissions. Kulldorf Statistics using SaTScan were applied to detect hotspots. Then, bivariate analysis was done using repeated values tabulation and analysis of covariance (ANCOVA). Inferential analysis was done using a mixed effect multivariable negative-binomial regression model, adjusting for spatiotemporal random effects.

**Results:**

A total of 2821 children were admitted more than once, giving a total of 6375 admissions. Of these 6375 admissions, 1866 were malnutrition-related, and 3.9% (109/2821) of the children with repeat admissions died. There was a seasonal pattern of re-admissions, peaking from May to July over the years. Hotspots were found in both the Northern and Southern areas of the KHDSS, while the areas near Kilifi Town were least affected. We found that disease severity was most likely associated with a malnutrition re-admission to the hospital.

**Conclusion:**

Disease severity was strongly associated with admission with malnutrition but its effect reduced after adjusting for the spatial and temporal random effects. Adjusting for clustering in space and in time (spatial-temporal) in models helps to improve the understanding of recurrent hospitalisations involving malnutrition.

**Electronic supplementary material:**

The online version of this article (10.1186/s40795-019-0296-5) contains supplementary material, which is available to authorized users.

## Background

Globally, malnutrition is a cause of 45% of childhood mortality, predominantly by potentiating morbidity due to common infections such as diarrhoea and pneumonia [1, 2]. Sub-Saharan Africa and South Asia remain the areas with the highest prevalence of malnutrition [2]. In Sub-Saharan Africa, malnutrition is a leading cause of death among children in marginalised populations [2, 3]. It remains a significant contributor to in-patient morbidity and mortality among children in rural areas in Kenya, despite efforts to overcome malnutrition [4–6]. When various infections occur, malnutrition complicates their management and increases their case fatality [7]. The case-fatality ratios for hospitalised severely-malnourished children typically range from 12% to more than 30% [2, 5, 8]. After discharge, an insufficient recovery period between illness episodes exacerbates malnutrition resulting in a vicious cycle. Malnutrition also majorly affects childhood development and increases the risk of non-communicable diseases and socioeconomic productivity in adulthood [1].

Undernutrition is a result of many determinants and not solely related to food insecurity. In Kenya, fundamental immediate and underlying causes of undernutrition have been identified to vary across the different age groups of 0–24 months and 25–59 months. Child’s birth size, breastfeeding patterns and acute morbidity experience have more impact on the younger age group compared to the underlying factors affecting the older age group [1, 9, 10]. Morbidity which is one of the immediate factors leading to undernutrition, often resulting in longer and repeated hospital admissions. Morbidity is therefore associated with increased inpatient costs to families and sustains impoverishment [9].

Because malnutrition is due to a complex interaction between different components, it is difficult to understand in a way that helps extend preventive and treatment strategies from current vertical interventions [1, 11]. Malnutrition has been reported to be spatially heterogeneous and also known to interact with various components of the environment, but little research has been done to integrate health, environmental and population data [12]. Up to now, a lack of detailed cohort and spatial data at homestead level had been a significant limitation for such spatial-temporal centred research of malnutrition in sub-Saharan Africa [11]. In this study, we use joint spatial-temporal models rather than mainly cross-sectional approaches [13] to give a different understanding of individual and contextual factors associated with recurrent hospital admissions involving malnutrition.

## Methods

### Study setting

**Fig. 1:**
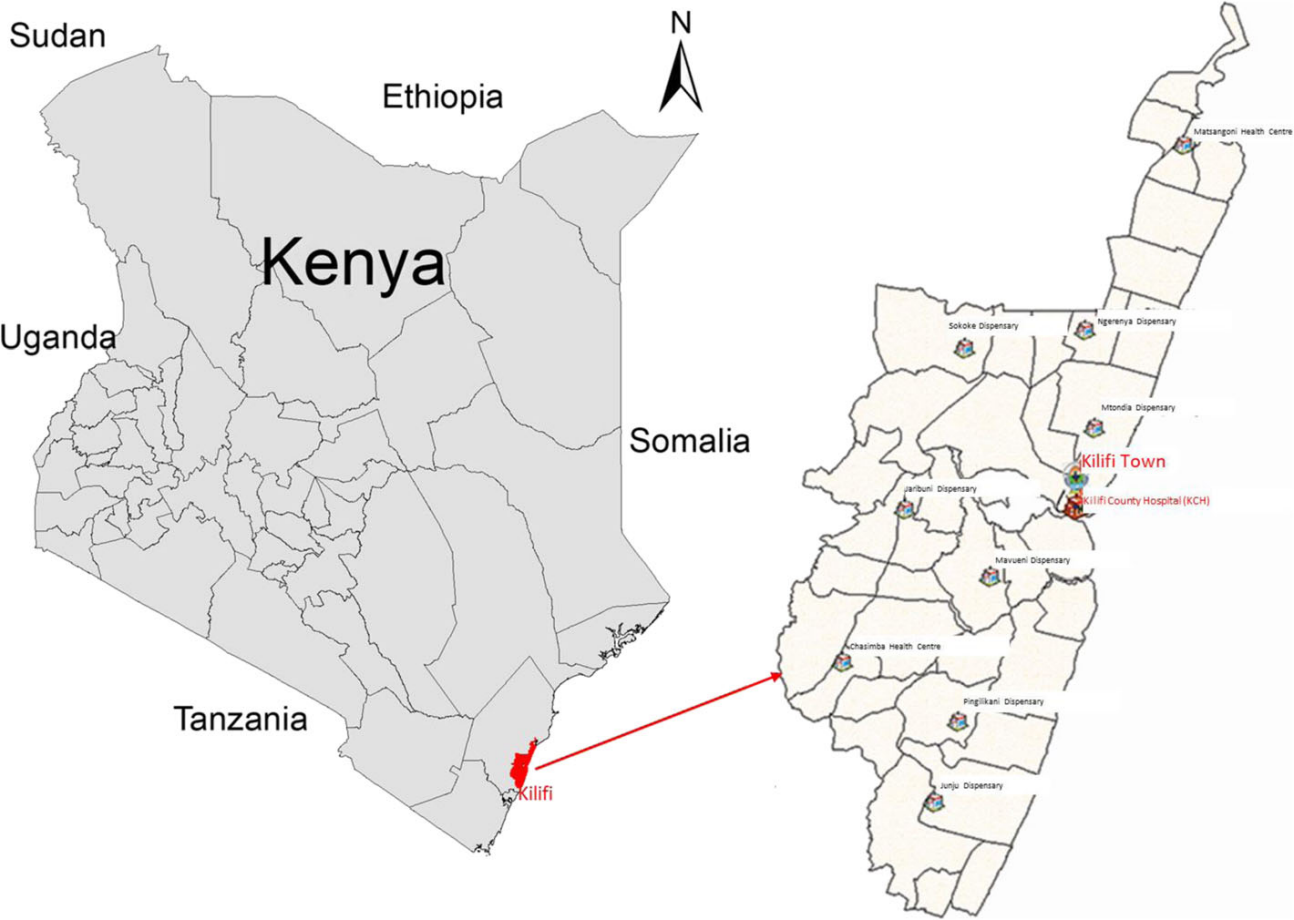
KHDSS Facilities. **a** The Kenyan map, showing the location of KHDSS, **b** The location of the KHDSS and the different dispensaries and the main county hospital

Secondary data analysis was conducted retrospectively on observational data from 2002 to 2015 of recurrent pediatric ward admissions to Kilifi County Hospital (KCH) in Kenya, including household data from a demographic surveillance. Kilifi County is located in the Coastal region of Kenya (shown in Fig. 1); it is a semi-arid area with subsistence farming being the main economic activity. KCH is the main referral hospital in Kilifi County with ~ 4000 pediatric admissions to KCH per year. KCH is in the middle of the Kilifi Health and Demographic Surveillance System (KHDSS) and captures resident children’s inpatient details at KCH pediatric ward. The KDHSS was set to monitor vital demographic indicators, mortality, migration and fertility in the area predominantly served by KCH.

The KHDSS covers 15 administrative locations and 40 sub-locations. The KHDSS area has approximately 280,000 residents. Data are collected three times a year from households to record deaths, migration and birth events. Mortality and morbidity events captured at the hospital are integrated with the population register [14]. The linkage of the surveillance data and admissions data is done real time with the matching of individuals at the point of admissions as explained in detail elsewhere [14]. The data is then de-identified by assigning a unique identifier and a corresponding person identifier for the KHDSS residents.

### Study participants

For this analysis, we included data from children admitted in KCH from April 2002 to December 2015, aged between 3 months and 5 years, residents of KHDSS who had more than one admission event. We excluded trauma events and children with a missing discharge outcome. The follow-up of the children started after the initial admission until the last admission before reaching 5 years of age (Additional file 1).

### Outcome and explanatory variables

The primary outcome of interest was the number of malnutrition-related readmissions. Malnutrition was defined using Weight for Height Z-score (WHZ) < − 2 or the presence of oedema at admission. Oedema is a clinical sign of undernutrition which may include swelling of the feet and skin [15]. The WHZ was calculated using the 2006 WHO child growth standards [16]. The weight of the children during admission was done using an electronic scale (Seca, Birmingham, UK) that has a weekly check for consistency [5, 17]. The heights of the children were measured with a stadiometer (Seca 215, Birmingham, UK) [18] except for children with less than 2 years and those who could not stand whose length was measured using a standard calibrated board.

The explanatory variables were selected with the guidance of the UNICEF malnutrition conceptual framework [1]. Child level variables, i.e. socio-demographic, anthropometric and clinical were selected as the immediate causes and the environmental variables as the underlying causes of malnutrition. We generated a composite variable for severe disease, defined as a child admitted with either gastroenteritis, Lower respiratory tract infection (LRTI), blood or Cerebrospinal fluid (CSF) culture positive, malaria and fever or meningitis [5]. The composite variable was generated from several severe co-morbidities that a child had during the readmission times. Environmental predictor variables were extracted from the Moderate Resolution Imaging Spectroradiometer (MODIS) under National Aeronautics and Space Administration’s (NASA’s) remote-sensing using the MODISTools and MODIS package in R© version 3.3.2 [19–21]. Estimates from Enhanced Vegetation Index (EVI) and Rainfall raster files were interpolated from values of the four nearest raster cells of each admission coordinate provided [12]. Additional covariates associated with malnutrition morbidity were identified by a total-sets analysis based on a generalised linear regression model and also those that have a biologically plausible relationship [22].

### Data quality

A team of field workers were trained on using the KHDSS surveillance system to do the matching of the data on real-time admissions. Each field worker has access to the web-based system following the matching procedures of the patients explained elsewhere [14]. Qualified medical and clinical officers enter the history and clinical examination of the patients in the web-app system after matching has been done by the field workers. Data quality checks on clinical measurements are implemented to ensure values entered into the system are within the normal biological range. The database has a daily backup, and each event or record entered into the system has a unique event identifier and an audit trail.

## Statistical methods

### Temporal exploratory data analysis

To explore the temporal patterns, the data were defined as a regular time series data using monthly time points of counts of malnutrition-related admissions. Augmented Dickey-Fuller unit-root was used to test for stationarity; the null hypothesis is there is no seasonality trend in the model. The stationary series was then examined on the Auto-Correlation Function (ACF) for the MA lags, and Partial Auto-Correlation Function (PACF) for the AR lags. This was followed by fitting combined Autoregressive integrated moving average (ARIMA) and the seasonal component to form the SARIMA models detailed in Additional file 2 [19, 23].

Seasonality was determined using the ACF and PCF with pointwise confidence intervals based on Bartlett’s formula. The Portmanteau test was used to confirm the significance of the seasonality. The Portmanteau test null hypothesis is that there is no serial correlation using the white noise under Pearson’s Chi-Square statistic [24]. This was followed by fitting a Seasonal ARIMA (SARIMA) model.

### Spatial exploratory analysis

The Global Moran Index was used to estimate the measure of sub-location spatial autocorrelation or randomness based on the observed cases of malnutrition admission. A distance matrix of the sub-locations centroids was used to calculate the Moran’s index [25, 26]. The Moran’s Index was calculated based on the central limit theory on the distribution of the index, which states that as the sample size increases the index tends to a normal distribution. The Moran Index is interpreted depending on the three outcomes; *I* > *E*(*I*) shows a positive autocorrelation implying clustering and *I* ≈ 0 shows no spatial autocorrelation and *I* < *E*(*I*) shows a negative autocorrelation implying a dispersed neighbouring value, where *E*(*I*) is the normally distributed population mean.

A window scanning over space was done comparing the observed versus the expected over the country using the SaTScan software. SaTScan applies the Kulldorff spatial scan statistic which imposes a circular window and calculates the likelihood of observing the events inside and outside the study area. The circle with the maximum likelihood is defined as the most likely cluster [27, 28]. This helps to determine local heterogeneity and clustering of malnutrition-related repeated admission to hospital to isolate the hot and cold spots in Kilifi County.

### Bivariate analysis

Our outcome was defined as the counts of malnutrition-related admissions, reflecting the burden and distribution of malnutrition and pattern of hospital utilisation. The data had a repeated event structure for the individuals over time. For the bivariate analysis, panel data tabulations were applied to investigate the between-individual variation and within-individual variation. This helps to understand the individual systematic difference and differences between individuals over time.

Repeated measures analysis of covariance (ANCOVA) was applied to compare the differences between the continuous predictors and the outcome.

### Inferential statistics

The malnutrition related morbidity was observed to follow a negative binomial distribution. Negative binomial is a generalisation of the Poisson distribution used to provide better epidemiological estimates of factors associated with malnutrition with repeated data [29, 30]. Secondly, the variance of the malnutrition-related admissions was higher than the mean, so a negative binomial distribution fitted our count data well [29, 31, 32]. Negative binomial spatial-temporal regression was applied to identify the spatial and temporal pattern of malnutrition admissions in Kilifi. The hypothesis tested was that readmission to hospital with malnutrition has spatial and temporal structure due to the individual level, and environmental covariates that underlie the risk. The count of admissions of the individuals was used as the temporal component and age was used as the exposure variable on our model. An autoregressive time series model was used to select the order for the temporal component to use in the spatial-temporal model.

Multivariable analysis was done using non-spatial, spatial and spatial-temporal models for admission specific and environmental variables with sub-location information.

### Model fit

We used the adapted Stochastic Partial Differential Equations (SPDE) in Integrated Nested Laplace approximation (INLA) for the spatial-temporal model, and the Markov chain Monte Carlo (MCMC) with Metropolis-Hastings algorithms approaches in OpenBUGS software from Medical Research Council (MRC) Biostatistics Unit [33] for spatial models using a Bayesian approach. STATA13™ was used to fit the multilevel models using Maximum Likelihood estimation. The advantage of Bayesian models over the MLE models is the combination of the prior information with the data through Bayes theorem to obtain posterior distributions. Convergence of the Bayesian Markov Chain Monte Carlo approach was monitored using the trace plots. For model comparison and best fit, we used the Deviance Information Criterion (DIC), where the smaller of the DIC was considered better taking into account the Pd. The pD is the difference of posterior mean deviance and deviance of posterior means which penalises for the parameters in the model [34, 35].

## Results

Over the 14 years, 2821 children under-5 years had at least two admissions in KCH, totalling 6375 admission events including the initial admission. Malnutrition-related admissions were identified in 1054/2821 (37% of children ever admitted) of the children. Of the 1054 children ever admitted with malnutrition, 76% (percentage within) of their re-admissions were malnutrition-related (Table 1). This shows that there are children who sometimes had a malnutrition re-admission and at other times a non-malnutrition related re-admission. Additionally, of the children with readmissions, 31.6% of the children had a positive malaria re-admission, and 74% of their re-admissions were reported to had malaria.

**Table 1:** Panel characteristics of the study population (Child Level Variables) for the period between 2002 and 2015

		Overall		
		Total Individuals	Overall Admissions (%)	Between *N* (% between; % within)^a^
Gender	Male	2821	3637 (57.0)	1583 (56.1;100)
	Female		2738 (43.0)	1238 (43.9;100)
Age group	3–23 months	2821	3846 (60.4)	2060 (73.0; 81.9)
	24-60 months		2527 (39.7)	1519 (53.9;74.6)
Malnutrition	NO	2821	4509 (70.7)	2282 (80.9;88.8)
	YES		1866 (29.3)	1054 (37.4;75.5)
Severe diseases	0	2821	2616 (41.4)	1709 (60.6;66.1)
	1		3434 (53.9)	2160 (76.6;71.5)
	2		307 (4.8)	284 (10.1;48.9)
	3		18 (0.3)	17 (0.6;47.1)
Severe anaemia	NO	2807	5695 (93.6)	2741 (97.7;96.0)
	YES		389 (6.4)	309 (11.0;57.1)
Hypoglycaemia	NO	2587	5173 (99.1)	2572 (99.4;99.5)
	YES		48 (0.9)	45 (1.7;62.5)
Malaria	NO	2805	4731 (78.2)	2375 (84.7;90.5)
	YES		1321 (21.8)	885 (31.6;74.1)
Diarrhoea	NO	2821	4992 (78.5)	2559 (90.7;85.6)
	YES		1366 (21.5)	1055 (37.4;59.8)
Menengitis	NO	2821	6295 (99.2)	2815 (99.8;99.3)
	YES		53 (0.8)	50 (1.8;50.7)
LRTI	NO	2821	4435 (69.9)	2441 (86.5;81.9)
	YES		1913 (30.1)	1285 (45.6;63.9)
Gastroenteritis	NO	2821	5391 (84.9)	2642 (93.7;89.8)
	YES		957 (15.1)	772 (27.4;58.3)
Transfused	NO	2819	5969 (94.1)	2768 (98.2;95.8)
	YES		376 (5.9)	302 (10.7;55.1)
Blood Culture	NO	2795	5901 (96.3)	2779 (99.4;96.9)
	YES		227 (3.7)	208 (7.4;48.7)
CSF Culture	NO	950	1185 (98.9)	942 (99.2;99.8)
	YES		13 (1.1)	12 (1.3;81.9)

NB: YES means positive test result for a given test, Between N tells us how many children had the specific characteristic of interest, % between tells us the fraction of the Total Individuals that had the characteristic, % within tells us the fraction of the re-admissions a child had the specified characteristic. Malnutrition (YES) – WHZ < -2 or Oedema, Severe anemia (YES)- Hemoglobin ≤ 5 g/dl, Hypoglycaemia (YES) - Blood glucose < 3.0 mmol/l, Malaria (YES) – parasite by microscopy fever, Meningitis (YES)- Final discharge diagnosis of meningitis; LRTI (Yes) – presence of Lower respiratory tract infections - Cerebrospinal fluid. ^a^ - one-way tabulation of counts of between and within individuals in repeated admissions data (panel data)

### Temporal exploration

The temporal analysis was fitted for monthly data for the period from April 2002 to December 2015. The time series autoregression order 1, integration order 0 and moving-average1 forming the ARIMA (1,0,1).

The AutoCorrelation Function (ACF) and Partial autocorrelation Function (PACF) of the transformed series using data from 2002 to 2015 showed peaks at different lag periods as shown in “Additional file 3”.

**Fig. 2:**
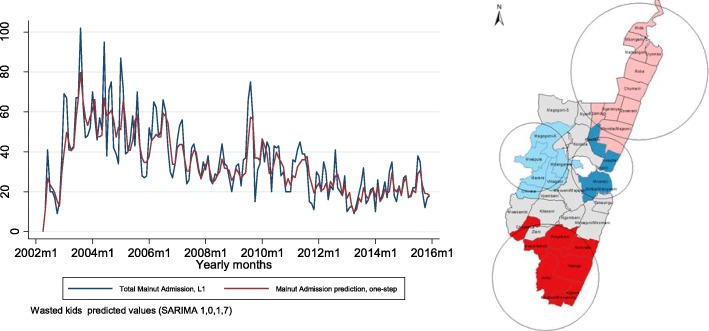
Time Series and Hotspot analysis results. **a** time series plot for monthly malnutrition recurrent admissions. **b** Hotspots and Coldspots of Malnutrition admissions between 2002 and 2015 in KHDSS

Significant serial correlation (seasonality) of months between May and July were observed for children admitted with malnutrition as shown in Fig. 2. This was confirmed with a significant (*p* < 0.001) Portmanteau test for white noise. Different peaks are observed, but in general, the malnutrition-related admissions decline over the period. The tables in “Additional file 3” shows the monthly counts of malnutrition admissions and mortality for the period between 2002 and 2015.

### Spatial exploration

A Global Moran’s I of 0.1 (*p*-value< 0.001, sd = 0.029) was observed showing a positive autocorrelation implying clustering. Based on the calculated Global Morans Index, we rejected the zero spatial autocorrelation hypothesis. The Kulldorff spatial scan statistic using SaTScan showed hotspots in the Northern and Southern areas of Kilifi urban area where the main hospital is based. Areas closer to the main hospital and Kilifi town were observed as cold spots of malnutrition-related admissions as shown in Fig. 2. Four of the five hot spots and cold spots were significant, the cluster (Mwapula, Marere, Magogoni-K, Mdangarani, Vinagoni, Chivara) was the one observed not to be significant for malnutrition-related admissions. The temporal clustering of malnutrition-related admissions (shown in “Additional file 3”), kept on shifting over the periods but similar regions were observed.

The age of the child at re-admission was used as the offset variable, and the count of malnutrition-related admissions was used as the temporal variable for more epidemiological intuitive results. The codes of the Integrated Nested Laplace approximation (INLA) approach and Multilevel modelling are in “Additional file 4”.

As shown on Table 2 three models were fitted, a multilevel model adjusting for the sub-location random effects, a spatial model and the spatial-temporal model using nested Laplace approximations which have better computational capability over the Markov Chain Monte Carlo approach (MCMC) [36]. The estimated mean coefficients are reported in Table 2 with a 95% confidence interval based on the Maximum Likelihood Estimation (MLE) approach and 95% credible intervals for the Bayesian approach.

**Table 2:** Non-spatial and spatial multivariable negative binomial models adjusted for duration of admission

Variables	Multi-level model		Spatial Structured and Unstructured Random Effects Model (INLA)	Spatial Structured and Unstructured and Temporal Random Effects Model
	Coefficients (95% C.I)	*p*-value	Mean (95% Cr .I)	Mean (95% Cr .I)
Location level variables				
EVI (	0.17 (-0.54;0.88)	0.644	0.07 (-0.58;0.70)	0.04 (-0.58;0.66)
Rainfall (mm)	0.03 (-0.03;0.09)	0.350	0.03 (-0.02;0.08)	0.03 (-0.02;0.08)
Child level variables				
Gender: Male	-0.15 (-0.25;-0.04)	0.006	-0.13 (-0.22;-0.04)	-0.13 (-0.22;-0.04)
Severe Disease				
1	0.41 (0.30;0.52)	<0.001	0.16 (0.06;0.25)	0.19 (0.10;0.28)
2	0.43 (0.18;0.69)	0.001	0.07 (-0.16;0.29)	0.15 (-0.07;0.37)
3	0.89 (0.06;1.71)	0.036	0.56 (-0.15;1.25)	0.53 (-0.16;1.18)
Total Number of Admissions	0.20 (0.17;0.23)	<0.001	0.2 (0.17;0.22)	0.07 (0.04;0.11)
DIC (pD)			10982.20 (21.46)	10656.28 (28.41)

In general, the spatial-temporal models had a lower DIC compared to non-spatial models but with extratemporal parameters. Bayesian spatial-temporal model using R-INLA was the model with the best fit with a DIC = 10,656.28 (pD = 28.41) which was the lowest compared to the spatial model which had a DIC =10,982.20 (pD = 21.46).

The spatial-temporal model showed consistent results with the non-spatial model except for the severe disease which changed. The final spatial-temporal negative binomial model was considered the “model of best fit” which caters well for over-dispersion and spatial-temporal confounding.

Adjusting for the spatial-temporal effects and other factors, males were less likely to have malnutrition re-admission (RR = 0.88 (Cr.I = 0.80–0.96) compared to the females. In the non-spatial model, the increase in the number of severe diseases increased the risk of malnutrition readmission. On the same note, the spatial-temporal effects, the levels 2 and 3 of severe diseases were insignificant. The environmental variables were not significantly associated with malnutrition re-admission in both the multilevel and spatial-temporal models.

**Fig. 3:**
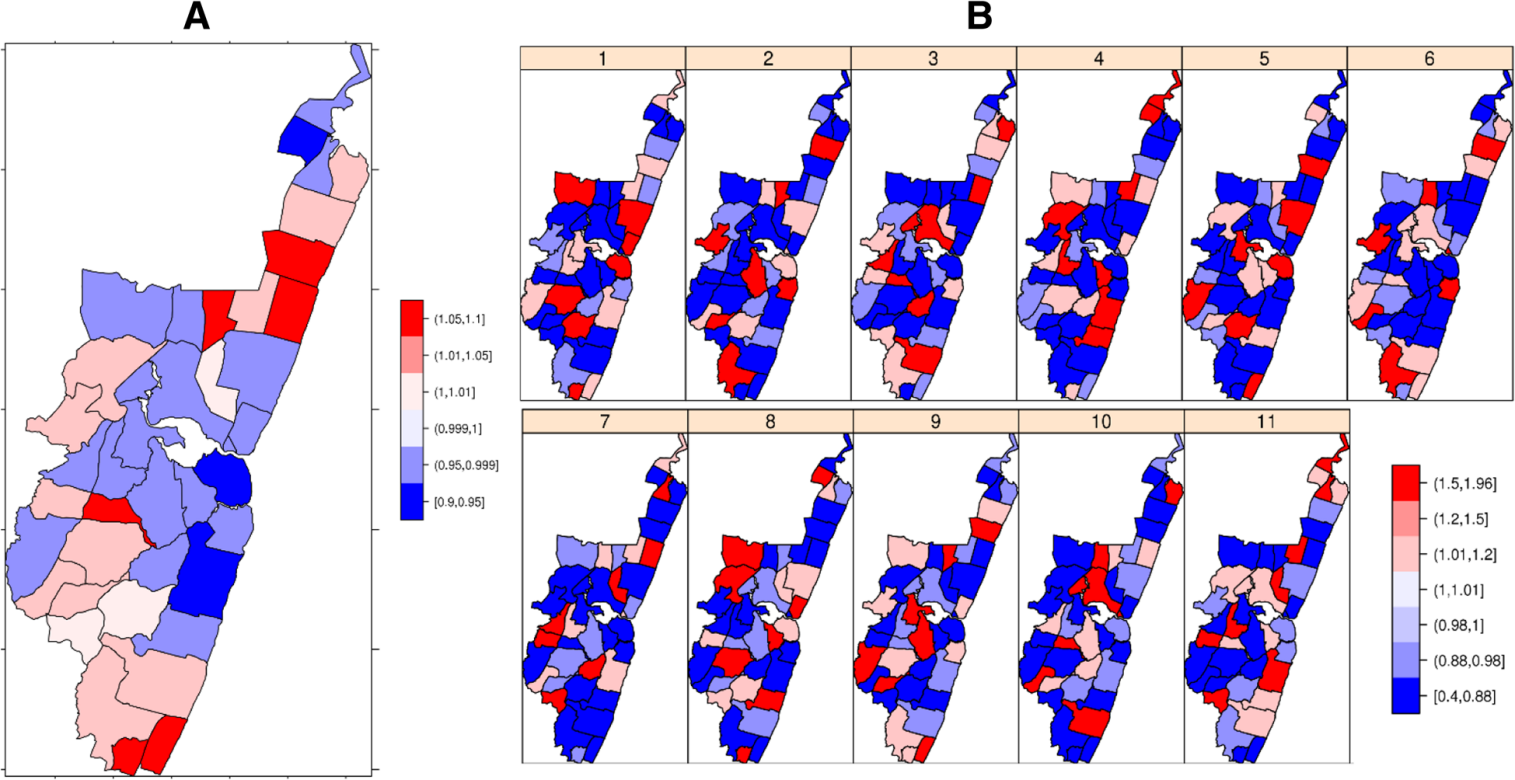
Bayesian modelling results. Malnutrition related hotspots (red shade) and cold spots (blue shade) in KHDSS 2002–2015, results from the Bayesian spatial-temporal; **a** Overall Model, **b** Temporal Variation of malnutrition re-admissions (re-admission 1 to 11)

Posterior temporal estimates of readmission were displayed on structured maps shown in Fig. 3 after running the spatial-temporal model. This shows the overall hot spots and cold spots of malnutrition readmission. Over the years, using Kuldorf Statistics (“Additional file 3”), the hotspots were consistent in the north and south of the creek. The readmission hotspots and cold spots were stable until the 6th readmission. The instability in the other readmission events could be due to a few children with these readmission events. Some sub-locations close to the Kilifi town and Kilifi County Hospital were identified as hotspots of readmission with malnutrition after adjusting for covariates. Areas with darker red have a higher risk of admission with malnutrition, and blue shades indicate a lower risk of a malnutrition admission. The plots in Appendix 7 (“Additional file 3”) shows that the parameters of the model fitted well since they follow a Gaussian distribution; thus the mean and the mode are equal.

## Discussion

This study describes the spatial-temporal admission of a group of children with repeated malnutrition-related re-admissions to Kilifi County Hospital admissions between 2002 and 2015. A seasonality of malnutrition-related re-admissions was observed, with peaks occurring in July when the rainy season was mainly observed. Emelda et al. reported a significant decline in malaria admissions at many hospitals in Kilifi. They reported this was observed against a background of elevating or regular non-malaria admissions and impervious to long-term rainfall throughout the surveillance period [37]. In Somalia, Kinyoki et al. observed a clear seasonal variation in wasting for children below 5 years due to variations in climate, food security, and infectious diseases. The peaks of malnutrition were observed during the dry season and were reported to have an elevated effect for the rainy season [37]. This can be used to explain the peaks of malnutrition-related admissions in Kilifi county hospital during the rainy season. However, the environmental factors were not associated with malnutrition readmissions.

Severe diseases, days of admission and rainfall were fit as covariates with malnutrition readmission as the outcome on our model. Severe disease was defined as the presence of either gastroenteritis, Lower respiratory tract infection (LRTI), blood and Cerebrospinal fluid (CSF) culture positive, positive malaria test examined by microscopy and fever or meningitis. In Somalia and Malawi, infections and geographical factors, including the Enhanced Vegetation Index were observed as critical drivers of malnutrition [13, 38]. Similarly, in our analysis children with a higher number of severe diseases were associated with an increased risk of the admission involving malnutrition. Contrary to the Somalia model, the EVI did not affect the risk of a malnutrition re-admission in the spatial-temporal model. However, overall, the mean EVI value (0.18) in Somalia was lower than the EVI value in Kilifi, Kenya (0.39).

In contrast to EVI, rainfall seasonality was associated with malnutrition admission which is similar to the time series analysis done by Karuri et al. that reported a seasonality during rainfall peaks for malaria admissions in the Kenya Coast [39]. This was different from a longitudinal malnutrition study done in Ethiopia where acute malnutrition did not have a significant seasonality [40]. Here, we combined both the spatial and temporal random effects and the environmental variables in our modelling approach. Thus, malnutrition could be possibly explained by the infections that occur during rainy seasons like diarrhoea or malaria.

## Conclusion

Sabrina et al. in 2014 review recommended the importance of combining spatial and temporal components in understanding the compounded phenomenon of malnutrition [11]. Though, with the different recommendations of spatial models to get an improved understanding of malnutrition; some of them require higher computing resources for an imbalanced and large dataset. In our model, we utilise a spatial-temporal approach which shows the importance of space and time in understanding the risk factors of malnutrition-related morbidity.

This study demonstrates that it is feasible to map episodes of repeated admission to hospital with malnutrition related morbidity at high spatial resolutions. The work has also utilised recently developed statistical tools together to develop spatial-temporal models that converged rapidly without loss of predictive accuracy. The contribution of infections provides a better understanding of the drivers of repeated admissions with malnutrition as a co-morbidity.

## Additional files

Additional file 1: **Appendix 1.** Flow diagram of data inclusion and exclusion. (DOCX 22 kb)

Additional file 2: **Appendix 2.** Time series and Bayesian model derivation. (PDF 144 kb)

Additional file 3: **Appendix 3.** Monthly malnutrition related admissions in Kilifi County Hospital between 2002 and 2015. **Appendix 4.** Autocorrelation Function (ACF) and Partial autocorrelation function (PACF) of the SARIMA model. **Appendix 5.** Shifting hotspots and colsdpots from 2002-2015 using Kulldorff statistics in SaTScan. **Appendix 7.** Negative Binomial Bayesian fit Diagnostic Plots. (DOCX 4710 kb)

Additional file 4: **Appendix 6.** R code for environmental data extraction and fitting the spatial temporal model. (DOCX 28 kb)

## Data Availability

The codes and MODIS data used and during the current study are available in the Github repository, under Additional file 4 or the online repository (https://github.com/Keniajin/BMC_nutrition_2019_spatial_temporal). The hospital admission datasets analyzed during the current study are not publicly available because it contains admission data of patient but are available from the corresponding author on reasonable request. Figures and the Appendix supplementary files are of my own except Fig. 1a shape file. The shape file for the Kenyan map is from GADM and the license is on https://gadm.org/license.html [accessed 25 April 2019]. The license states that “GADM license The data are freely available for academic use and other non-commercial use. Redistribution, or commercial use, is not allowed without prior permission. Using the data to create maps for academic publishing is allowed”.

## Abbreviations

ACF: Autocorrelation Function
ANCOVA: Analysis of Covariance
AR: Auto Regressive
ARIMA: AutoRegressive Integrated Moving Average
CSF: Cerebrospinal fluid
DIC: Deviance Information Criterion
EVI: Enhanced Vegetation Index
INLA: Integrated Nested Laplace Approximation
KCH: Kilifi County Hospital
KHDSS: Kilifi Health and Demographic Surveillance System
LRTI: Lower Respiratory Tract Infection
MCMC: Markov Chain Monte Carlo
MLE: Maximum Likelihood Estimation
MODIS: Moderate Resolution Imaging Spectroradiometer
PACF: Partial autocorrelation function
SARIMA: Seasonal AutoRegressive Integrated Moving Average
SPDE: Stochastic Partial Differential Equations
UNICEF: United Nations Children’s Fund
WHZ: Weight for Height Z Scores
