# Supplementary material for: A space-time analysis of recurrent malnutrition-related hospitalisations in Kilifi, Kenya for children under-5 years
Source: BMC Nutr. 2019 Jun 4;5:32. doi: 10.1186/s40795-019-0296-5 (PMC7050923; doi:10.1186/s40795-019-0296-5)
Supplement: Supplementary file 1 — Appendix 1. Flow diagram of data inclusion and exclusion. (DOCX 22 kb) [file 40795_2019_296_MOESM1_ESM.docx]

Appendix 1:

**24,584 (32,210 admission events)**

11,541 events aged <3 or >61 months
1,170 Trauma Events
163 Events Missing outcome or Z-score
268 missing person ID

**14,961 (19,068 admission events)**

12,661 single admission events

**2,821 (6,375 admission events)**

**597 (1,866 malnutrition admission events)**

**2,224 (4,509 Non malnutrition admission events)**
