# Supplementary material for: A space-time analysis of recurrent malnutrition-related hospitalisations in Kilifi, Kenya for children under-5 years
Source: BMC Nutr. 2019 Jun 4;5:32. doi: 10.1186/s40795-019-0296-5 (PMC7050923; doi:10.1186/s40795-019-0296-5)
Supplement: Supplementary file 2 — Appendix 2. Time series and Bayesian model derivation. (PDF 144 kb) [file 40795_2019_296_MOESM2_ESM.pdf]

Appendix 2: Model Derivation

SARIMA MODEL

In general the seasonal autoregressive moving average model is defined as

$$ARIMA(p,d,q)\ (P,D,Q)_s$$

$\uparrow$ Non seasonal $\uparrow$ *seasonal* where s is the number periods per season.

SARIMA is an $ARIMA(p,d,q)$ model whose residuals at ℓ_t are $ARIMA(P,D,Q)$

NEGATIVE BINOMIAL MODEL

Our outcome was defined as the counts of malnutrition related admissions until the person with the highest number of malnutrition related admissions. Secondly, the variance of the malnutrition related admissions was higher than the mean so a negative binomial distribution fitted our count data well(29,31,32). The morbidity outcome data followed a negative binomial distribution; which is a generalisation of the Poisson distribution used to provide better epidemiological estimates of factors associated with malnutrition morbidity (29) . The negative binomial spatial-temporal model was applied to identify the spatial and temporal pattern of malnutrition admissions in Kilifi (32,41). We included children with at least two admissions events for the spatial-temporal negative binomial model due to computational requirements. The readmissions would also help investigate vulnerability due to malnutrition morbidity.

The observed admission data used for the malnutrition morbidity modelling, there were 2,820 individuals indexed by $i = 1,2,3,....,n_t = 2820$ who lived in one of the 40 sub-locations indexed by $j = 1,2,3,....,q = 40$ with the repeated admissions visits ranging from time $t = 1,2,3,...., p = 11$ (42).

The definition of the negative binomial spatial model is

$$y_{ijt} \left| \psi_{ijt}, \underset{\sim}{\Omega} \sim \pi\left(y_{ijt} \left| \psi_{ijt}; \underset{\sim}{\Omega} \right.\right)\right.$$

$\underset{\sim}{\Omega}$ (used as Ω thereafter) is a vector of parameters to be estimated and ψ_{ijt} is the linear predictor. Where $y_{ijt} = \left[y_{ijt} \right]_{n\times p}$ are the observed admission data which follow a negative binomial distribution of the form $y_{ijt} \sim NegBinomial(p_{ijt}, r_{ijt})$ (43)

The subscript for p_{ijt} and r_{ijt} maybe dropped for some of the derivations for convenience.

$$p(y=\left[y_{ijt}\right]\mid p,r)=\binom{y_{ijt}-1}{r-1}p^{r}(1-p)^{y_{ijt}};y\geq r,r=1,2,3...;0<p<1$$

Equation 1

where $r=\frac{1}{\theta}$ and $p_{ijt}=\frac{1}{1+\theta\mu_{ijt}}$

Using our data and y is the number of admissions from each individual i, r is the number of successful malnutrition admissions from sub-location j at admission t and p is the probability of a malnutrition admission.

The conditional mean is $E\left(y_{ijt}\mid p,r\right)=\frac{r(1-p_{ijt})}{p_{ijt}}$ and conditional variance $\mathrm{var}(y_{ijt}\mid p,r)=\frac{r(1-p_{ijt})}{p_{ijt}^{2}}$.

Taking the natural logs from **Equation 1** above,

$$=\ln\binom{y_{ijt}-1}{r-1}+r\ln\left(p_{ijt}\right)+y_{ijt}\ln(1-p_{ijt})$$

Equation 2

And in general the exponential family form of a negative binomial is expressed as a member of the generalized linear model which has a link function and a cumilant as shown below;

$$f\left(y;p,r\right)=\exp\left\{\underbrace{y_{ijt}\ \ln\left(1-p_{ijt}\right)}_{\text{link function}}+\underbrace{r\ln\left(p_{i}\right)}_{\text{cumilant}}+\ln\binom{y_{ijt}-1}{r-1}\right\}$$

Equation 3

Using $r=\frac{1}{\theta}$ and $p_{ijt}=\frac{1}{1+\theta\mu_{ijt}}$ from equation 3 above, and replace in equation 4 , this is gives

$$=\ln\left(\frac{\Gamma\left(y_{ijt}\right)}{\Gamma\left(y_{ijt}-\frac{1}{\theta}\right)\Gamma\left(\frac{1}{\theta}\right)}\right)+\frac{1}{\theta}\ln\left(\frac{1}{1+\theta\mu_{ijt}}\right)+y_{ijt}\ln\left(1-\frac{1}{1+\theta\mu_{ijt}}\right)$$

Equation 4

Then after taking the exponents, this can be expressed as

$$= \exp\left\{ \ln\Gamma\left(y_{ijt}\right) + y_{ijt}\ln\left(\frac{\theta\mu_{ijt}}{1+\theta\mu_{ijt}}\right) - \ln\Gamma\left(y_{ijt} - \frac{1}{\theta}\right) + \frac{1}{\theta}\ln\left(\frac{1}{1+\theta\mu_{ijt}}\right) - \ln\Gamma\left(\frac{1}{\theta}\right)\right\}$$

Equation 5

Since the Poisson-Gamma joint distribution marginal is a Negative-Binomial, then the expression is as follows (43);

$$= \exp\left\{ c_0\left(\mu_{ijt},\theta\right) + \ln\Gamma\left(y_{ijt}\right) - \ln\Gamma\left(y_{ijt} - \frac{1}{\theta}\right) - \ln\Gamma\left(\frac{1}{\theta}\right)\right\}$$

Equation 6

Where

$$c_0\left(\mu_{ijt},\theta\right) = y_{ijt}\ln\left(\frac{\theta\mu_{ijt}}{1+\theta\mu_{ijt}}\right) + \frac{1}{\theta}\ln\left(\frac{1}{1+\theta\mu_{ijt}}\right) = y_{ijt}\ln\left(\frac{\theta\mu_{it}}{1+\theta\mu_{it}}\right) - \frac{1}{\theta}\ln\left(1+\theta\mu_{it}\right)$$

Where $\mu_{ijt} = \exp\left(\psi_{ijt}\right) = \log\left(E_{ijt}\right) + x_{ijt}\underset{\sim}{\beta} + \underset{\sim}{\phi}_j + \underset{\sim}{\vartheta}_j + \underset{\sim}{\gamma}_t + \varepsilon_{ijt}$ with E_{ijt} as the ages of the children and is the exposure time variable, x_{ijt} are the covariates design matrix, $\underset{\sim}{\beta}$ vector of fixed coefficients. The INLA modelled latent variables for structured ($\underset{\sim}{\phi}_j$), unstructured ($\underset{\sim}{\vartheta}_j$) space and time ($\underset{\sim}{\gamma}_t$) and the error terms ε_{ijt} based on the fitted and the predicted values.

Our spatial temporal model was fitted assuming the age of the child as the exposure variable.

Under multilevel maximum likelihood estimation, the negative binomial form was expressed as (31,43)

$$L\left(\mu_{ijt} \mid y_{ijt},\theta\right) = \prod_{i=1}^{n_t} \exp\left\{ c_0\left(\mu_{ijt},\theta\right) + \ln\Gamma\left(y_{ijt} - \frac{1}{\theta}\right) - \ln\Gamma\left(y_{ijt}\right) - \ln\Gamma\left(\frac{1}{\theta}\right)\right\}$$

Equation 7

.

The log likelihood is obtained by taking the log of the likelihood

$$\ell\left(\mu_{ijt} \mid y_{ijt}, \theta\right) = \exp\left[\sum_{i=1}^{n_t}\left\{c_0\left(\mu_{ijt}, \theta\right) + \ln\Gamma\left(y_{ijt} - \frac{1}{\theta}\right) - \ln\Gamma\left(y_{ijt}\right) - \ln\Gamma\left(\frac{1}{\theta}\right)\right\}\right]$$

Equation 8

Considering values with the parameters , substituting μ_{ijt} with the linear predictor and $c_0\left(\mu_{ijt}, \theta\right)$, the negative binomial log-likelihood, in terms of covariates, the model coefficients, can be expressed as:

$$\propto \exp\left[\sum_{i=1}^{n_t}\left\{y_{ijt}\ln\left(\frac{\theta\exp(\psi_{ijt})}{1+\theta\exp(\psi_{ijt})}\right) - \frac{1}{\theta}\ln\left(1+\theta\exp(\psi_{ijt})\right)\right\}\right]$$

Equation 9

Thus our model can be implemented using GLM with the link function being (43,44)

$$y_{ijt}\ln\left(\frac{\theta\exp(\psi_{ijt})}{1+\theta\exp(\psi_{ijt})}\right)$$

Since the prior distribution of μ_{ijt} is a multivariate normal with a mean zero and a $\sum_{q\times q} = \sum_{40\times 40}$ variance matrix, $\mu_{ijt} = MVN\left(0, \sum_{40\times 40}\right)$. The likelihood contribution for the jth sub location is obtained by integrating μ_{ijt} out of the joint probability density $f\left(y_{ijt} \mid \mu_{ijt}, \theta\right)$

$$L\left(\Omega, \Sigma, \theta\right) = \left(2\pi\right)^{-\frac{q}{2}}\left|\Sigma\right|^{-\frac{1}{2}}\int f\left(y_{ijt} \mid \mu_{ijt}, \theta\right)\exp\left(\mu_{ijt}^{'}\,\Sigma^{-1}\frac{\mu_{ijt}}{2}\right)d\mu_{ijt}$$

Equation 10

The above equation has no closed form and thus approximation method is used for Maximum likelihood estimation.

The spatial temporal Bayesian model is defined as follows as explained above

$$Posterior[\,p(parameters \mid data)] \propto Likelihood \times \Pr iors$$

The full conditional for our model can be expressed as

$$p(\Omega \mid y_{ijt}) \propto L(y_{ijt} \mid \Omega) \times p(\underset{\sim}{\beta}_k) \times p(\underset{\sim}{\phi}_j \mid \tau_c) \times p(\underset{\sim}{\vartheta}_j \mid \tau_h) \times p(\underset{\sim}{\gamma}_t \mid \tau_e)$$

$$= L(y_{ijt} \mid \Omega) \times p(\underset{\sim}{\beta}_k) \times p(\underset{\sim}{\phi}_j \mid \tau_c) \times p(\tau_c) \times p(\underset{\sim}{\vartheta}_j \mid \tau_h) \times p(\tau_h) \times p(\underset{\sim}{\gamma}_t \mid \tau_e)$$

Equation 11

The prior for the beta coefficients for k-1 fixed predictors in the model is assumed to be $\underset{\sim}{\beta}_k \overset{iid}{\sim} N(\mu_\beta, \sigma_\beta{}^2)$ therefore

$$p(\beta_k) = \frac{1}{\sqrt{2\pi\sigma_\beta^2}} \exp\left[-\frac{1}{2}\left(\frac{\beta_k - \mu_\beta}{\sigma_\beta}\right)^2\right]$$

Equation 12

The unstructured random effects in Gibbs sampling $p(\underset{\sim}{\vartheta}_j \mid \tau_h)$, where

$\vartheta_j \overset{iid}{\sim} N\left(0, \frac{1}{\tau_h}\right)$ Therefore

$$p(\vartheta_j \mid \tau_h) = \frac{1}{\sqrt{\frac{2\pi}{\tau_h}}} \exp\left[-\frac{1}{2}\left(\frac{\vartheta_j - 0}{\frac{1}{\sqrt{\tau_h}}}\right)^2\right]$$

Equation 13

with a Gamma function prior used $\tau_h \sim Gamma(\alpha_h, \beta_h)$ therefore (33)

$$p(\tau_h) = \frac{(\beta_h)^{\alpha_h}}{\Gamma(\alpha_h)} \tau_h^{\alpha_h - 1} \exp(-\beta_h \tau_h), \alpha_h > 0; \beta_h > 0$$

Equation 14

Therefore the unstructured random effects prior in BUGS is expressed as;

$$p\left(\vartheta_j \mid \tau_h\right) \times p\left(\tau_h\right) \propto \tau_h^{\alpha_h - 1} \exp(-\beta_h \tau_h) \times \exp\left[-\frac{1}{2}\left(\frac{\vartheta_j - 0}{\frac{1}{\sqrt{\tau_h}}}\right)^2\right]$$

Equation 15

In BUGS, a CAR prior is used for the structured spatial random effects (45) $\phi_j \sim CAR\left(\tau_c\right)$ anda CAR prior given by prior is given as $\phi_j \mid \phi_i, j \neq i, \tau_c \sim N\left(\frac{\phi_j}{\overline{\phi_j}}, \frac{1}{\tau_c m_j}\right)$. Therefore

$$p\left(\phi_j \mid \tau_c\right) = \frac{1}{\sqrt{\frac{2\pi}{\tau_c m_j}}} \exp\left[-\frac{1}{2}\left(\frac{\phi_j - \frac{\phi_j}{\overline{\phi_j}}}{\sqrt{\frac{1}{\tau_c m_j}}}\right)^2\right]$$

Equation 16

Hence the likelihood of the neighbouring sub locations is given as

$p\left(\phi_j \mid \tau_c\right) \propto \exp\left\{-\frac{\tau_c}{2}\sum_{i=1}^{n_t} w_{ij}\left(\phi_j - \phi_i\right)^2\right\}$ where w_{ij} denotes the adjacency matrix and shows that the sub location \$j\$ is a neighbour of sub location \$i\$ and \$m_j\$ is the number of neigbours for sub location j. A conjugate hyper prior of $\tau_c \sim Gamma\left(\alpha_c, \beta_c\right)$ is assumed

$$p(\tau_c) = \frac{\left(\beta_c\right)^{\alpha_c}}{\Gamma\left(\alpha_c\right)} \tau_c^{\alpha_c - 1} \exp\left(-\beta_c \tau_c\right), \alpha_c > 0; \beta_c > 0$$

Equation 17

Therefore;

$$p\left(\phi_j \mid \tau_c\right) \times p(\tau_c) \propto \exp\left\{-\frac{\tau_c}{2}\sum_{i=1}^{n} w_{ij}\left(\phi_j - \phi_i\right)^2\right\} \tau_c^{\alpha_c - 1} \exp\left(-\beta_c \tau_c\right)$$

Equation 18

In INLA,the structured temporal component $\underset{\sim}{\phi}_j = f_{spat}\left(s_j\right)$,a Besag CAR prior was used similar to CAR prior given by prior is given as $s_j \mid s_i, j \neq i, \tau_c \sim N\left(\frac{1}{m_j}\sum_{j\sim i} s_j, \frac{1}{\tau_c m_j}\right)$.

Where $\phi_j = \left(\phi_1, \phi_2, \phi_3, \ldots., \phi_{40}\right)$, m_j is the number of neighbours of sub location j , $j \sim i$ indicates that the two sub locations j and i are neighbours (46).

The temporal component used in the spatial temporal model was defined as $\underset{\sim}{\gamma}_t = f_{temp}\left(y_t\right) \sim AR\left(1\right)$ first order autoregressive model with normal first term prior

$$y_t = \rho y_{t-1} + \varepsilon_t$$

$y_1 = N\left(0, \frac{1}{\tau_e\left(1-\rho^2\right)}\right)$ $\left|\rho_t\right| < 1$ for stationarity $\varepsilon_t \sim N\left(0, \tau_e^{-1} = \sigma^2\right)$ is the white noise process (46,47).

Therefore auto regressive parameter is expressed as

$$p\left(\rho \mid \sigma_t\right) = \frac{1}{\sqrt{2\pi\sigma_t^2}} \exp\left(\frac{-(y_t - \rho y_{t-1})^2}{2\sigma_t^2}\right)$$

Equation 19

Finally combining the likelihood and the prior to obtain our posterior distribution as shown in Equation 11

$$p\left(\Omega \mid y_{ijt}\right) = \exp\left[\sum_{i=1}^{n_t}\left\{y_{ijt} \ln\left(\frac{\theta \exp(\psi_{ijt})}{1+\theta\exp(\psi_{ijt})}\right) - \frac{1}{\theta}\ln\left(1+\theta\exp(\psi_{ijt})\right)\right\}\right] \times$$

$$\frac{1}{\sqrt{2\pi\sigma_\beta^2}} \exp\left[-\frac{1}{2}\left(\frac{\beta_k - \mu_\beta}{\sigma_\beta}\right)^2\right] \times \tau_h^{\alpha_h - 1} \exp(-\beta_h \tau_h) \times \exp\left[-\frac{1}{2}\left(\frac{\vartheta_j - 0}{\frac{1}{\sqrt{\tau_h}}}\right)^2\right]$$

$$\times \exp\left\{-\frac{\tau_c}{2}\sum_{i=1}^{n} w_{ij}\left(\phi_j - \phi_i\right)^2\right\} \tau_c^{\alpha_c - 1} \exp\left(-\beta_c \tau_c\right) \times \frac{1}{\sqrt{2\pi\sigma_t^2}} \exp\left(\frac{-(y_t - \rho y_{t-1})^2}{2\sigma_t^2}\right)$$

Equation 20

This combination has no closed form; estimation is used to estimate the parameters. To solve for the parameters, we use the adapted Stochastic Partial Differential Equations (SPDE) in INLA, and the MCMC with Metropolis- Hastings algorithms approaches in WinBUGS (29,31,33).
