# Supplementary material for: A space-time analysis of recurrent malnutrition-related hospitalisations in Kilifi, Kenya for children under-5 years
Source: BMC Nutr. 2019 Jun 4;5:32. doi: 10.1186/s40795-019-0296-5 (PMC7050923; doi:10.1186/s40795-019-0296-5)

**Appendix 3: Monthly malnutrition related admissions**

| Year | Jan | Feb | Mar | Apr | May | Jun | Jul | Aug | Sep | Oct | Nov | Dec | Total |
| --- | --- | --- | --- | --- | --- | --- | --- | --- | --- | --- | --- | --- | --- |
| 2002 |  |  |  | 12 | 41 | 20 | 20 | 17 | 9 | 15 | 37 | 69 | 240 |
| 2003 | 67 | 41 | 41 | 43 | 67 | 67 | 102 | 63 | 47 | 48 | 52 | 70 | 708 |
| 2004 | 57 | 46 | 57 | 51 | 95 | 38 | 71 | 75 | 42 | 39 | 34 | 87 | 692 |
| 2005 | 71 | 39 | 40 | 45 | 58 | 43 | 70 | 46 | 28 | 27 | 28 | 52 | 547 |
| 2006 | 46 | 65 | 62 | 48 | 50 | 66 | 60 | 42 | 30 | 27 | 34 | 46 | 576 |
| 2007 | 53 | 56 | 37 | 24 | 26 | 42 | 44 | 38 | 30 | 27 | 35 | 31 | 443 |
| 2008 | 38 | 26 | 24 | 29 | 28 | 36 | 44 | 34 | 33 | 27 | 20 | 27 | 366 |
| 2009 | 33 | 34 | 23 | 36 | 37 | 66 | 75 | 58 | 15 | 31 | 34 | 46 | 488 |
| 2010 | 35 | 45 | 42 | 20 | 43 | 42 | 43 | 28 | 30 | 20 | 20 | 20 | 388 |
| 2011 | 36 | 35 | 41 | 45 | 39 | 39 | 32 | 15 | 14 | 11 | 30 | 28 | 365 |
| 2012 | 20 | 35 | 30 | 16 | 23 | 21 | 41 | 22 | 20 | 18 | 28 | 10 | 284 |
| 2013 | 12 | 13 | 9 | 14 | 19 | 24 | 32 | 14 | 16 | 21 | 22 | 10 | 206 |
| 2014 | 26 | 15 | 19 | 25 | 17 | 30 | 35 | 18 | 15 | 23 | 18 | 27 | 268 |
| 2015 | 28 | 17 | 18 | 22 | 22 | 38 | 35 | 20 | 12 | 17 | 18 | 13 | 260 |
| Total | 522 | 467 | 443 | 430 | 565 | 572 | 704 | 490 | 341 | 351 | 410 | 536 | 5,831 |

**Appendix 4: ACF and PACF**

**Appendix 5: Shifting hotspots and colsdpots from 2002-2015.**

**Appendix 7: Diagnostic Plots**


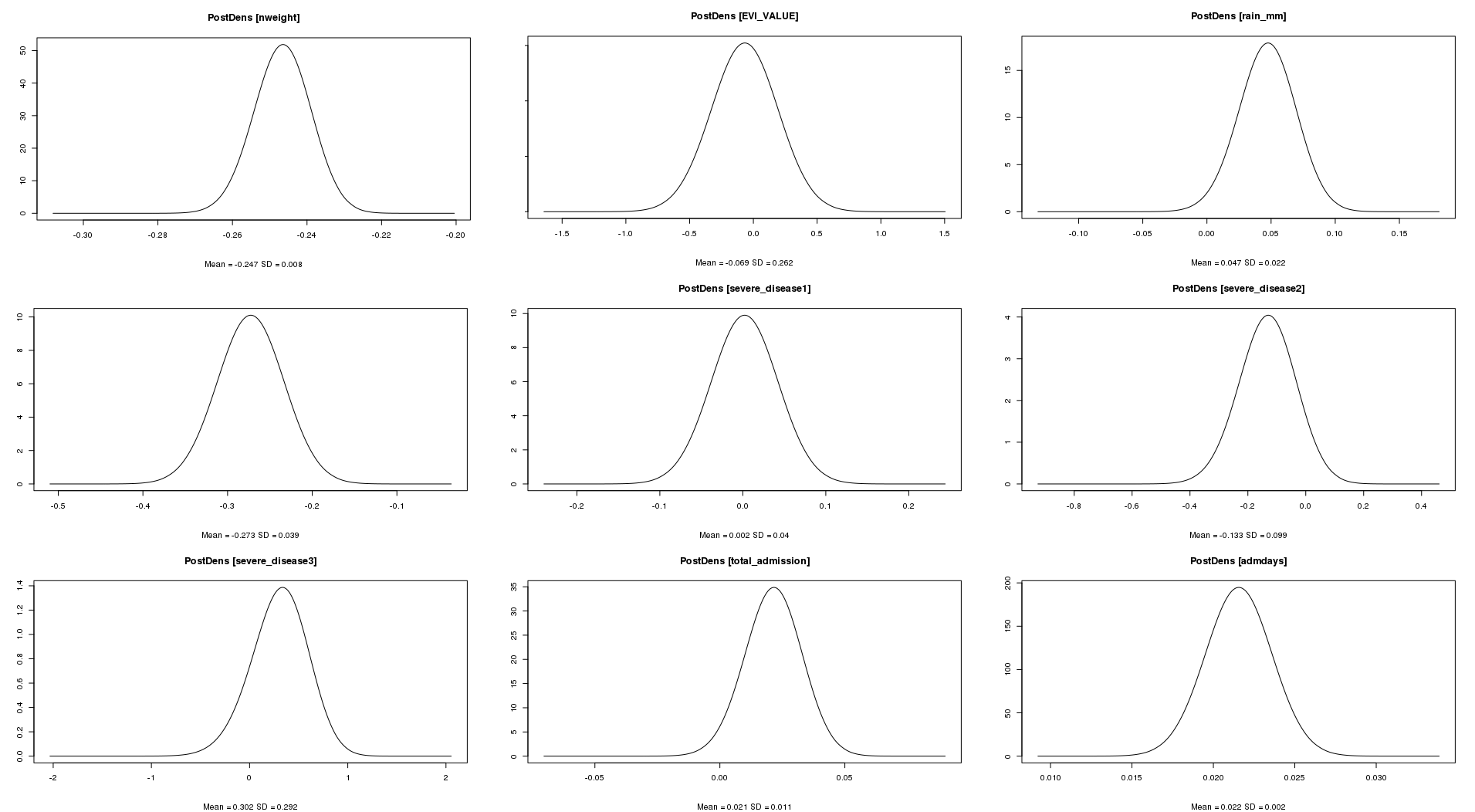

Supplement: Supplementary file 3 — Appendix 3. Monthly malnutrition related admissions in Kilifi County Hospital between 2002 and 2015. Appendix 4. Autocorrelation Function (ACF) and Partial autocorrelation function (PACF) of the SARIMA model. Appendix 5. Shifting hotspots and colsdpots from 2002-2015 using Kulldorff statistics in SaTScan. Appendix 7. Negative Binomial Bayesian fit Diagnostic Plots. (DOCX 4710 kb) [file 40795_2019_296_MOESM3_ESM.docx]
