# Supplementary material for: A space-time analysis of recurrent malnutrition-related hospitalisations in Kilifi, Kenya for children under-5 years
Source: BMC Nutr. 2019 Jun 4;5:32. doi: 10.1186/s40795-019-0296-5 (PMC7050923; doi:10.1186/s40795-019-0296-5)
Supplement: Supplementary file 4 — Appendix 6. R code for environmental data extraction and fitting the spatial temporal model. (DOCX 28 kb) [file 40795_2019_296_MOESM4_ESM.docx]

Appendix 6 - Analysis Code

Contents

[Code 1 - Extract weather data 1](#_Toc7419848)

[Code 2: INLA Spatial Temproral Models 2](#_Toc7419849)

[Code 3: Temporal graphs for Spatial Temporal 9](#_Toc7419850)

## Code 1 - Extract weather data

Script for extracting MODIS vegetation indices for analysis

set.seed(1221223)
library(MODIS)
library(dplyr)
library(foreign)
library(tidyr)

##read in the data with locations from KHDSS
## 1km by 1km latitude and longitude for the 1 by 1 km locations
## this file contains the dat[1] latitude and dat[2] longitude for the 1km by 1km locations
dat <- foreign::read.dta("data/KHDSS_1km_1km.DTA")

#' \\\_Extract the rainfall data process_\\\ #
#'#'\\_________Starting the extract of data_______________________\\


##identify the folder with the monthly tiff data for EVI
## the files were downloaded earlier and saved
## https://modis.gsfc.nasa.gov/data/dataprod/mod13.php
##https://www.rdocumentation.org/packages/MODIS/versions/1.1.5/topics/runGdal

## alink that can help https://conservationecology.wordpress.com/2014/08/11/bulk-downloading-and-analysing-modis-data-in-r/

#'install https://www.gdal.org/
#'library(MODIS)
#'runGdal(product="MOD13Q1",begin=as.Date("01/02/2002",format = "%d/%m/%Y") ,
#' end = as.Date("31/12/2015",format = "%d/%m/%Y"),extent="Kenya")
#'
#' 'getTile("Kenya") for tile specific
#' ,tileH = 21:22,tileV = 8:9
#'

#'(this process takes some time and needs 30 GB of space free to generate data for the whole admission period
vi <- preStack(path = "modis/monthly_data/", pattern = "*.tif$")

### stack the data to data frame
s <- stack(vi)
s <- s * 0.0001 # Rescale the downloaded Files with the scaling factor (from modis) EVI

#'#'\\_________extracting for the 1km by 1km data_________\\
# And extract the mean value for our point from before.
# First Transform our coordinates from lat-long to to the MODIS sinus Projection
## method='bilinear' used for extraction
## If 'simple' values for the cell a point falls in are returned.
## If 'bilinear' the returned values are interpolated from the values of the four nearest raster cells.

sp <- SpatialPoints(coords = cbind(dat[2], dat[1]),
 proj4string = CRS("+proj=longlat +datum=WGS84 +ellps=WGS84") )
sp <- spTransform(sp, CRS(proj4string(s)))
dataExtract <- raster::extract(s, sp , df=T, method="bilinear") # Extract the EVI
write.csv(dataExtract , "data/modis_data_2001_2015.csv")

#'@_______________________________________________________________
#' \\\_End Extract data process_\\\ #
#'


####
#'#'\\_________Extracting the rainfall data_________\\
###Extracting the rainfall data
##identify the folder with the monthly tiff data for EVI
vi2 <- preStack(path = "modis/rainfall/", pattern = "resampledchirps-v2.0.20*")

### stack the data to data frame
si2 <- stack(vi2)

#'#'\\_________extracting for the admissions data_________\\
sp2_b <- SpatialPoints(coords = cbind(dat[2], dat[1]),
 proj4string = CRS("+proj=longlat +datum=WGS84 +ellps=WGS84"))
sp2_b <- spTransform(sp2_b, CRS(proj4string(si2)))
dataExtractAdm2 <- raster::extract(si2, sp2_b , df=T, method="bilinear") # Extract the rainfall
write.csv(dataExtractAdm2 , "dataExtractADM_rainfall.csv")
#'@_______________________________________________________________

## Code 2: INLA Spatial Temproral Models

This code is for running INLA Spatial models

set.seed(1221223)
 rm(list = ls())

 library(R2WinBUGS)
 ##Mapping
 library(rgeos)
 library(maptools)
 library("ggplot2")
 library(broom) ## for converting a map to a data frame
 #library(glm2)
 #library(ResourceSelection) ## for hosmer and lemeshow testing
 library(dplyr)
 library(INLA)
 library(spdep)
 ## coloring the spplot
 library(colorspace)

 ###reading and exporting the shape file
 ## shape file available upon request
 kilifi_sub <- maptools::readShapePoly ( "data/kilif_sub_loc_Shape/DSS_subloc_Arc.shp",
 IDvar="Adj_ID", proj4string=CRS("+proj=longlat +ellps=clrk66"))

 temp <- spdep::poly2nb(kilifi_sub)
 nb2INLA("data/kilif_sub_loc_Shape/DSS_subloc_Arc.graph", temp)
 klf.adj <- paste(getwd(),"/data/kilif_sub_loc_Shape/DSS_subloc_Arc.graph",sep="")

 ### load the admissions data
 ## data available upon request
 admData <- read.csv("data/morbidity.csv")
 admData$rain_mm <- admData$rain_mm/50
 admData$severe_disease <- factor(admData$severe_disease , levels=c(0,1,2,3))
 admData <- admData %>% mutate(gender2= ifelse(gender==1 ,0,1 ))

 admData$gender2 <- factor(admData$gender2 , levels=c(0,1) )
 admData$gender <- admData$gender2
 admData2 <- admData %>% dplyr::select(Adj_ID , sublocation,mnth, nagem, gender ,
 severe_disease ,
 cumulitive_count,cumulitive_time , EVI_VALUE ,count_adm ,rain_mm,
 total_admission ,admdays ,nweight ,yr)

 ###generate othe variables to be used within INLa
 admData2$Adj_ID2 <- admData2$Adj_ID
 admData2$Adj_ID3 <- admData2$Adj_ID
 admData2$count_adm2 <- admData2$count_adm
 admData2$count_adm3 <- admData2$count_adm
 admData2$count_adm4 <- admData2$count_adm
 admData2$count_adm5 <- admData2$count_adm
 admData2$count_adm6 <- admData2$count_adm
 admData2$count_adm7 <- admData2$count_adm
 admData2$EVI_VALUE2 <- admData2$EVI_VALUE
 admData2$rain_mm2 <- admData2$rain_mm
 admData2$nagem2 <- admData2$nagem
 admData2$severe_disease2 <- as.factor(admData2$severe_disease)
 admData2$mnth2 <- admData2$mnth
 admData2$nweight2 <- admData2$nweight
 admData2$admdays2 <- admData2$admdays

 ###defining the priors
 prior.iid = c(1,0.01)
 prior.besag = c(1,0.001)
 initial.iid = 4
 initial.besag = 3

 #' \\\_Model_1_\\\ #
###############MOdel 1 #######################################################
 ## spatial unstructured
 formulaUH0 <- cumulitive_count ~ EVI_VALUE + rain_mm + gender + severe_disease +
 total_admission + admdays + nweight +
 f(Adj_ID, model = "iid",prior="normal",param=c(0, 0.001) , initial = 1)

 resultUH0 <- inla(formulaUH0,family="nbinomial",
 data=admData2, control.compute=list(dic=TRUE,cpo=TRUE),E=log(nagem) ,
 control.predictor = list(compute = TRUE))

##summary in 3 decimal places
 summary(resultUH0)
 exp(resultUH0$summary.fixed)
 write.csv(data.frame(resultUH0$summary.fixed), "results1_14504_36.csv")

pdresultUH0 <- resultUH0$dic$p.eff


 #' \\\_Model_1_\\\ #
 #' #

 #' \\\_Model 1B\\\ #
 ###############MOdel 1B#######################################################
 ### spatial model structured and unstrustured without
 ### to comapare with Winbugs
 formulaUHB <- cumulitive_count ~ EVI_VALUE + rain_mm + gender + severe_disease +
 total_admission + admdays + nweight +
 f(Adj_ID, model = "bym" ,graph=klf.adj , scale.model=TRUE,
 hyper=list(prec.unstruct=list(prior="loggamma",param=c(0.0111,0.001)),
 prec.spatial=list(prior="loggamma",param=c(0.0011,0.001))))


 resultUHB <- inla(formulaUHB,family="nbinomial",
 data=admData2, control.compute=list(dic=TRUE,cpo=TRUE),E=log(nagem)
 ,control.predictor(compute=TRUE))
 summary(resultUHB)
 pdresultUHB <- resultUHB$dic$p.eff #25.03
 exp(resultUHB$summary.fixed)
 write.csv(data.frame(resultUHB$summary.fixed), "results2_14498.08.csv")

 #write.csv(data.frame(resultUHB$summary.fixed), "results_20.05_under5_10700.63.csv")

 ####The computation of the posterior mean for the random effects ð is performed in two
 # steps as we have more than one parameter:
 # we extract the marginal posterior distribution for each element of the random effect
 csi <- resultUHB$marginals.random$Adj_ID[1:40]

 ## then apply the exponential transformation and calculate the posterior mean for each of them using the lapply function.
 zeta <- lapply(csi,function(x) inla.emarginal(exp,x))
 ##define the cut offs for your risk ratio
 zeta.cutoff <- c(0.9, 0.95, 0.999 ,1.0,1.01,1.05, 1.1)

 #Transform zeta in categorical variable
 cat.zeta <- cut(unlist(zeta),breaks=zeta.cutoff,
 include.lowest=TRUE )

 #Create a dataframe with all the information needed for the map
 maps.cat.zeta <- data.frame(unique(admData2$Adj_ID), cat.zeta=cat.zeta)

 #Add the categorized zeta to the kilifi spatial polygon
 ##
 data.kilifi <- attr(kilifi_sub, "data")
 attr(kilifi_sub, "data") <- merge(data.kilifi, maps.cat.zeta,
 by.x="Adj_ID" , by.y="unique.admData2.Adj_ID.")

 ## mapping the risk ratio
 #spplot(obj=kilifi_sub, zcol= "cat.zeta", col.regions=gray(seq(0.9,0.1,length=4)), asp=1)
 spplot(obj=kilifi_sub, zcol= "cat.zeta",col.regions=diverge_hsv(8), scales=list(draw = TRUE), asp=1)


#' \\\_Model 2\\\ #
###############MOdel 2#######################################################
### spatial model structured and unstrustured with the temporal component included
### fitting model 1
admData2$nagem_int <- as.integer(admData2$nagem)

formulaUH <- cumulitive_count ~ EVI_VALUE + rain_mm + gender + severe_disease + total_admission + admdays + nweight +
 f(Adj_ID, model = "bym" ,graph=klf.adj , scale.model=TRUE,hyper=list(prec.unstruct=list(prior="loggamma",param=c(0.0111,0.001)),
 prec.spatial=list(prior="loggamma",param=c(0.0011,0.001)))) + f(count_adm, model = "ar1")

# f(count_adm, model = "ar1", replicate = Adj_ID3)
resultUH <- inla(formulaUH,family="nbinomial",
 data=admData2, control.compute=list(dic=TRUE,cpo=TRUE),E=log(nagem_int)
 ,control.predictor(compute=TRUE))
summary(resultUH)
pdresultUH <- resultUH$dic$p.eff #35.50
exp(resultUH$summary.fixed)
write.csv(data.frame(resultUH$summary.fixed), "nm_results2_13640.2.csv")


####The computation of the posterior mean for the random effects ð is performed in two
# steps as we have more than one parameter:
# we extract the marginal posterior distribution for each element of the random effect
csi <- resultUH$marginals.random$Adj_ID[1:40]

## then apply the exponential transformation and calculate the posterior mean for each of them using the lapply function.
zeta <- lapply(csi,function(x) inla.emarginal(exp,x))
##define the cut offs for your risk ratio
zeta.cutoff <- c(0.83,0.9, 0.95, 0.999 ,1.0,1.01,1.05, 1.1 ,1.2)

#Transform zeta in categorical variable
cat.zeta <- cut(unlist(zeta),breaks=zeta.cutoff,
 include.lowest=TRUE )

#Create a dataframe with all the information needed for the map
maps.cat.zeta <- data.frame(unique(admData2$Adj_ID), cat.zeta=cat.zeta)

#Add the categorized zeta to the kilifi spatial polygon
##
data.kilifi <- attr(kilifi_sub, "data")
attr(kilifi_sub, "data") <- merge(data.kilifi, maps.cat.zeta,
 by.x="Adj_ID" , by.y="unique.admData2.Adj_ID.")

## mapping the risk ratio

png(filename=paste0("figure4A","img.png") , width = 19.45 , height = 22.40 , units = "cm" , res=300)

spplot(obj=kilifi_sub, zcol= "cat.zeta",col.regions=diverge_hsv(8), scales=list(draw = TRUE), asp=1)
dev.off()

### temporal graph

plot( resultUH, plot.fixed.effects = TRUE, constant=FALSE,
 plot.lincomb = TRUE,
 plot.random.effects = TRUE,
 plot.hyperparameters = TRUE,
 plot.predictor = TRUE,
 plot.q = TRUE,
 plot.cpo = TRUE,
 single = TRUE)

 plot( resultUH, plot.fixed.effects = TRUE , constant=FALSE,plot.cpo = F,single =F)

save.image("stModel.RDA")

#' \\\_Model 3\\\ #
#' ###############MOdel With variables changing over time#######################################################
#### Fitting a SPATIAL Temporal Model
formulaUH2b <- cumulitive_count ~ EVI_VALUE + gender +
 severe_disease + total_admission + rain_mm + admdays + nweight +
 f(Adj_ID, model = "bym" ,graph=klf.adj , scale.model=TRUE,
 hyper=list(prec.unstruct=list(prior="loggamma",param=c(0.001,0.001)),
 prec.spatial=list(prior="loggamma",param=c(0.1,0.01))))+
 f(EVI_VALUE2 , count_adm2, model = "iid") +
 f(rain_mm2 , count_adm3, model = "iid") +
 f(nweight2 , count_adm5, model = "iid") +
 f(admdays2 , count_adm6, model = "iid") +
 f( count_adm7, model = "ar1")

### added due to heissan values errors
##https://groups.google.com/forum/#!topic/r-inla-discussion-group/rTdjAnILdnM
resultUH2b <- inla(formulaUH2b,family="nbinomial",
 data=admData2, control.compute=list(dic=TRUE),control.predictor(compute=TRUE) ,
 control.inla = list(tolerance = 1e-20, h = 1e-08),E=log(nagem))
pdresultH2 <- resultUH2b$dic$p.eff #447.2864


summary(resultUH2b)
pdresultUH2b <- resultUH2b$dic$p.eff
write.csv(data.frame(resultUH2b$summary.fixed), "resultsST_10296.73.csv")

csi2 <- resultUH2b$marginals.random$Adj_ID[1:40]

## then apply the exponential transformation and calculate the posterior mean for each of them using the lapply function.
zeta2 <- lapply(csi2,function(x) inla.emarginal(exp,x))

##define the cut offs for your risk ratio
zeta.cutoff2 <- c(0.8,0.99, 1.0,1.001,1.1, 1.2)


#Transform zeta in categorical variable
cat.zeta2 <- cut(unlist(zeta2),breaks=zeta.cutoff2,
 include.lowest=TRUE)

#Create a dataframe with all the information needed for the map
maps.cat.zeta2 <- data.frame(unique(admData2$Adj_ID), cat.zeta2=cat.zeta2)

#Add the categorized zeta to the kilifi spatial polygon
##
data.kilifi2 <- attr(kilifi_sub, "data")
attr(kilifi_sub, "data") <- merge(data.kilifi2, maps.cat.zeta2,
 by.x="Adj_ID" , by.y="unique.admData2.Adj_ID.")

## mapping the risk ratio
spplot(obj=kilifi_sub, zcol= "cat.zeta2",col.regions=diverge_hsv(8), scales=list(draw = TRUE), asp=1)

### temporal graph
plot( resultUH2b, plot.fixed.effects = TRUE , constant=FALSE,plot.cpo = F,single =F)
plot( resultUH2b, plot.fixed.effects = TRUE, constant=FALSE,
 plot.lincomb = TRUE,
 plot.random.effects = TRUE,
 plot.hyperparameters = TRUE,
 plot.predictor = TRUE,
 plot.q = TRUE,
 plot.cpo = TRUE,
 single = TRUE)

## Code 3: Temporal graphs for Spatial Temporal

This code is for plotting the temporal graphs for the repeated admission,we fit the model in a loop for each admission and also as a temporal effect on INLA

### this is a loop fitting a model for each admission count and saving the graph for each admisison count
for (i in unique(admData2$count_adm)) {
 rm(list = ls())
 ###reading and exporting the shape file
 kilifi_sub <- maptools::readShapePoly ( "data/kilif_sub_loc_Shape/DSS_subloc_Arc.shp",
 IDvar="Adj_ID", proj4string=CRS("+proj=longlat +ellps=clrk66"))

 temp <- spdep::poly2nb(kilifi_sub)
 nb2INLA("data/kilif_sub_loc_Shape/DSS_subloc_Arc.graph", temp)
 klf.adj <- paste(getwd(),"/data/kilif_sub_loc_Shape/DSS_subloc_Arc.graph",sep="")

 ### load the admissions data
 admData <- read.csv("data/morbidity.csv")
 admData$rain_mm <- admData$rain_mm/50
 admData$severe_disease <- factor(admData$severe_disease , levels=c(0,1,2,3))
 admData <- admData %>% mutate(gender2= ifelse(gender==1 ,0,1 ))

 admData$gender2 <- factor(admData$gender2 , levels=c(0,1) )
 admData$gender <- admData$gender2
 admData2 <- admData %>% dplyr::select(Adj_ID , sublocation,mnth, nagem, gender ,
 severe_disease ,
 cumulitive_count,cumulitive_time , EVI_VALUE ,count_adm ,rain_mm,
 total_admission ,admdays ,nweight ,yr)

 ###
 admData2$Adj_ID2 <- admData2$Adj_ID
 admData2$Adj_ID3 <- admData2$Adj_ID
 admData2$count_adm2 <- admData2$count_adm
 admData2$count_adm3 <- admData2$count_adm
 admData2$count_adm4 <- admData2$count_adm
 admData2$count_adm5 <- admData2$count_adm
 admData2$count_adm6 <- admData2$count_adm
 admData2$count_adm7 <- admData2$count_adm
 admData2$EVI_VALUE2 <- admData2$EVI_VALUE
 admData2$rain_mm2 <- admData2$rain_mm
 admData2$nagem2 <- admData2$nagem
 admData2$severe_disease2 <- as.factor(admData2$severe_disease)
 admData2$mnth2 <- admData2$mnth
 admData2$nweight2 <- admData2$nweight
 admData2$admdays2 <- admData2$admdays


 admData2x <- admData2 %>% filter(count_adm==i)
 formulaUH <- cumulitive_count ~ EVI_VALUE + rain_mm +
 gender + severe_disease + total_admission + admdays + nweight +
 f(Adj_ID, model = "bym" ,graph=klf.adj , scale.model=TRUE,
 hyper=list(prec.unstruct=list(prior="loggamma",param=c(1,0.001)),
 prec.spatial=list(prior="loggamma",param=c(1,0.001))))

# f(count_adm, model = "ar1", replicate = Adj_ID3)
 resultUH <- inla(formulaUH,family="nbinomial",
 data=admData2x, control.compute=list(dic=TRUE,cpo=TRUE),E=log(nagem)
 ,control.predictor(compute=TRUE))

#summary(resultUH)

#exp(resultUH$summary.fixed)
 write.csv(data.frame(exp(resultUH$summary.fixed)), paste0(i,"_results2_10504.53.csv"))
####The computation of the posterior mean for the random effects ð is performed in two
# steps as we have more than one parameter:
# we extract the marginal posterior distribution for each element of the random effect
csi <- resultUH$marginals.random$Adj_ID[1:40]

## then apply the exponential transformation and calculate the posterior mean for each of them using the lapply function.
zeta <- lapply(csi,function(x) inla.emarginal(exp,x))
##define the cut offs for your risk ratio
zeta.cutoff <- c(0.9, 0.95, 0.999 ,1.0,1.01,1.05, 1.1)

#Transform zeta in categorical variable
cat.zeta <- cut(unlist(zeta),breaks=zeta.cutoff,
 include.lowest=TRUE )

#Create a dataframe with all the information needed for the map
maps.cat.zeta <- data.frame(unique(admData2$Adj_ID), cat.zeta=cat.zeta)

#Add the categorized zeta to the kilifi spatial polygon
##
data.kilifi <- attr(kilifi_sub, "data")
attr(kilifi_sub, "data") <- merge(data.kilifi, maps.cat.zeta,
 by.x="Adj_ID" , by.y="unique.admData2.Adj_ID.")

## mapping the risk ratio
png(filename=paste0("temp_", i,"_count.png") , width = 15.47 , height = 17.57 , units = "cm" , res=72)
spplot(obj=kilifi_sub, zcol= "cat.zeta",col.regions=diverge_hsv(8), scales=list(draw = TRUE), asp=1)
dev.off()
}


## here we fit an AR1 model with INLA and extract the plots for each timepoint
## allow for an interaction between space and time,
#which would explain differences in the time trend of malnutrition related admissions for different areas,
### Type III interaction - used in this paper to report
##Type III combines the unstructured temporal effect ????t and the spatially structured main effect ui

ID.area.int <- admData2$Adj_ID
ID.year.int <- admData2$count_adm
temporalModel3 <- cumulitive_count ~ EVI_VALUE + gender +
 severe_disease + rain_mm + admdays + nweight +
 f(Adj_ID, model = "bym" ,graph=klf.adj , scale.model=TRUE,
 hyper=list(prec.unstruct=list(prior="loggamma",param=c(0.001,0.001)),
 prec.spatial=list(prior="loggamma",param=c(0.1,0.01)))) +
 f( count_adm, model = "ar1") + f(ID.year.int,model="iid", group=ID.area.int,
 control.group=list(model="besag",
 graph=klf.adj))

result_tM3 <- inla(temporalModel3,family="nbinomial",
 data=admData2, control.compute=list(dic=TRUE),control.predictor(compute=TRUE) ,
 control.inla = list(tolerance = 1e-20, h = 1e-08),E=log(nagem_int))


delta.intIII <- data.frame(delta=exp(result_tM3$summary.random$ID.year.int[,2]),tempC=rep(1:11, each = 40),
 ID.area=result_tM3$summary.random$ID.year.int[,1])
delta.intIII.matrix <- matrix(delta.intIII[,1], 40,11,byrow=FALSE)
rownames(delta.intIII.matrix)<- delta.intIII[1:40,3]

save.image("st_model3.RDA")


cutoff.interaction <- c(0.50,0.880, 1.0, 1.3,1.9,2.5,3.4,7.4)
data.klf <- attr(kilifi_sub, "data")
delta.intIII.factor <- data.frame(NAME=data.klf$Adj_ID)
for(i in 1:11){
 delta.factor.temp <- cut(delta.intIII.matrix[,i],breaks=cutoff.interaction,include.lowest=TRUE )
 delta.intIII.factor <- cbind(delta.intIII.factor,delta.factor.temp)
}
colnames(delta.intIII.factor)<- c("NAME",seq(1,11))

# *** Code for Figure 7.6
attr(kilifi_sub, "data") <- data.frame(data.klf, intIII=delta.intIII.factor)
trellis.par.set(axis.line=list(col=NA))

png(filename=paste0("temp_","img.png") , width = 25.47 , height = 27.57 , units = "cm" , res=300)

spplot(obj=kilifi_sub, zcol=c("intIII.1","intIII.2","intIII.3",
 "intIII.4", "intIII.5","intIII.6",
 "intIII.7", "intIII.8","intIII.9",
 "intIII.10","intIII.11"),
 col.regions=diverge_hsv(8),
 names.attr=seq(1,11),main="")
dev.off()

## code for fitting with interaction 2
### Type II interaction
##Type II combines the structured temporal main effect and unstructured interactions
#f(ID.area.int,model="iid", group=ID.year.int,control.group=list(model="ar1"))
## interaction 2
ID.area.int <- admData2$Adj_ID
ID.year.int <- admData2$count_adm
temporalModel2 <- cumulitive_count ~ EVI_VALUE + gender +
 severe_disease + total_admission + rain_mm + admdays + nweight +
 f(Adj_ID, model = "bym" ,graph=klf.adj , scale.model=TRUE,
 hyper=list(prec.unstruct=list(prior="loggamma",param=c(0.001,0.001)),
 prec.spatial=list(prior="loggamma",param=c(0.1,0.01)))) +
 f( count_adm, model = "ar1") + f(ID.area.int,model="iid", group=ID.year.int,control.group=list(model="ar1"))


result_tM2 <- inla(temporalModel2,family="nbinomial",
 data=admData2, control.compute=list(dic=TRUE),control.predictor(compute=TRUE) ,
 control.inla = list(tolerance = 1e-20, h = 1e-08),E=log(nagem))

###
delta.intII <- data.frame(delta=exp(result_tM2$summary.random$ID.area.int[,2]),
 tempC=rep(1:11, each = 40) ,ID.area=result_tM2$summary.random$ID.area.int[,1])
delta.intII.matrix <- matrix(delta.intII[,1], 40,11,byrow=FALSE)
rownames(delta.intII.matrix)<- delta.intII[1:40,3]


# Check the absence of spatial trend for (intII)
# cutoff.interaction <- c(-1,-0.01,0.01,1)
cutoff.interaction <- c(0.20, 0.50,0.70, 0.999 ,1.0,1.01, 1.1,1.4,1.7)
data.klf <- attr(kilifi_sub, "data")
delta.intII.factor <- data.frame(NAME=data.klf$Adj_ID)
for(i in 1:11){
 delta.factor.temp <- cut(delta.intII.matrix[,i],breaks=cutoff.interaction,include.lowest=TRUE)
 delta.intII.factor <- cbind(delta.intII.factor,delta.factor.temp)
}
colnames(delta.intII.factor)<- c("NAME",seq(1,11))

# *** Code for Figure 7.5
attr(kilifi_sub, "data") <- data.frame(data.klf, intII=delta.intII.factor)
trellis.par.set(axis.line=list(col=NA))

spplot(obj=kilifi_sub, zcol=c("intII.1","intII.2","intII.3",
 "intII.4", "intII.5","intII.6",
 "intII.7", "intII.8","intII.9",
 "intII.10","intII.11"),
 col.regions=diverge_hsv(8),
 names.attr=seq(1,11),main="")
